# Revealing the diversity of a once small taxon: the genus *Selenoribates* (Acari, Oribatida, Selenoribatidae)

**DOI:** 10.3897/zookeys.312.5478

**Published:** 2013-06-24

**Authors:** Tobias Pfingstl

**Affiliations:** 1 Bermuda Institute of Ocean Sciences Inc. (BIOS), 17 Biological Lane, St. George’s GE 01, Bermuda; 2 Institute of Zoology, University Graz, Universitaetsplatz 2, 8010 Graz, Austria

**Keywords:** Bermuda, intertidal, juvenile instars, *Selenoribates*, biogeography

## Abstract

Three new intertidal oribatid species, *Selenoribates elegans* **sp. n.**, *Selenoribates quasimodo* **sp. n.** and *Selenoribates satanicus* **sp. n.** are described from the archipelago of Bermuda. *Selenoribates elegans* **sp. n.** is characterized by its slender body shape, *Selenoribates quasimodo* **sp. n.** possesses a hunchback in lateral view and *Selenoribates satanicus* **sp. n.** exhibits two horn-like projections on its anterior gastronotic region. Based on these new findings, the number of *Selenoribates* species doubled at once and the distribution of this genus, formerly limited to the Mediterranean and the Red Sea, includes now occurrences in the Atlantic and Indo-pacific Ocean as well. The morphology of *Selenoribates quasimodo* **sp. n.** and *Selenoribates satanicus* **sp. n.** deviates conspicuously from the other known members of *Selenoribates*, thus indicating that not only the number of species but also the anatomy of this genus is more diverse than formerly supposed. Nymphs of *Selenoribates quasimodo* **sp. n.** show an interesting case of ontogenetic neotrichy, with gastronotic setae being duplicated with each moult.

## Introduction

The family of Selenoribatidae represents a group of littoral oribatid mites. These mites are air-breathing terrestrial organisms, but they have managed to colonize marine associated habitats and are now exclusively confined to intertidal zones of coastal areas (Pfingstl 2013). They show a transoceanic distribution and occur on shores of the tropics and subtropics (e.g. Schuster 1989, Procheş and Marshall 2001). At present, this family consists of seven genera, namely *Arotrobates* Luxton, 1992, *Carinozetes* Pfingstl and Schuster, 2012, *Psednobates* Luxton, 1992, *Rhizophobates* Karasawa & Aoki, 2005, *Schusteria* Grandjean, 1968, *Selenoribates* Strenzke, 1961 and *Thalassozetes* Schuster, 1963. *Rhizophobates* and *Schusteria* have been subject to taxonomic debates (e.g. Karasawa and Aoki 2005, Pfingstl and Schuster 2012) and the discreteness of some members of these genera is still unclear. The other genera are yet well delimited and the same applies to the genus type *Selenoribates*. Strenzke (1961) described the first species of this taxon, *Selenoribates foveiventris*, then Grandjean (1966) discovered *Selenoribates mediterraneus*, and finally Abd-El-Hamid (1973) added the third species, *Selenoribates ghardaqensis*. Since that time no further species have been detected and not even a single record of the already known species has been published. Accordingly the genus *Selenoribates*, with only three species, was supposed to be a small taxon with a restricted distribution in the Mediterranean and the Red Sea. The descriptions of the known species provided ample data on the morphology of the adults (Strenzke 1961, Grandjean 1966, Abd-El-Hamid 1973) but only one author (Grandjean 1966) described the nymphs of *Selenoribates mediterraneus*. Concerning the ecology and biology, virtually nothing is known about these species.

In the course of a recent study on intertidal oribatid mites from Bermuda, three new *Selenoribates* species could be discovered and this finding changes biogeographic and morphological aspects of this genus dramatically. Therefore this paper describes the morphology of the three new species, modifies the distribution pattern and tries to answer the question why the genus *Selenoribates* has vanished into thin air for more than forty years.

## Material and methods

Intertidal algae growing on sandy and rocky substrate, as well as on roots of the black mangrove (*Avicennia germinans*) were collected on the archipelago of Bermuda during low tide and afterwards put in a Berlese-Tullgren apparatus for the extraction of mites. For investigation in transmitted light all animals were stored in ethanol (70% or pure ethanol), then heated in lactic acid (80°C for about 20 minutes) and afterwards embedded in BERLESE mountant. Observations, photographs and drawings were made with an Olympus BH-2 Microscope equipped with a drawing attachment. Image stacks were obtained by an Olympus E1 digital camera and layered with the Combine ZP software. Inscriptions of drawings were done according to Grandjean (1966, 1968).

## Results

### Family Selenoribatidae Schuster, 1963

#### Genus Selenoribates

Strenzke, 1961

http://species-id.net/wiki/Selenoribates

##### Remarks.

The following diagnosis summarizes the characters provided by Strenzke (1961), Grandjean (1966), Abd-El-Hamid (1973) and includes the characters of the present descriptions.

Small sized (198–308 × 119–185 µm) intertidal mites. Cerotegument granular. Interlamellar setae short or minute. Lamellar ridges present but short. Sensillus flagelliform and long. Pedotectum I small but robust, pedotectum II absent. Notogaster with 14 pairs of setae, *c_3_* absent. Obvious depressions, variable in number and shape, present on anterior part of notogaster. Two median epimeral cavities present. Epimeral setal formula 1-0-1-1. Genital plates with three-four pairs of setae. Aggenital setae absent. Two-three pairs of adanal setae and one-three pairs of anal setae. Legs monodactylous; claws with one or two proximoventral teeth. Juveniles plicate with large centrodorsal plate.

#### Selenoribates quasimodo sp. n.

urn:lsid:zoobank.org:act:CA3698B8-B235-4EAA-8DC3-E3F1DDDF7D84

http://species-id.net/wiki/Selenoribates_quasimodo

##### Type locality.

Bermuda: Coney Island, 32°21'30"N, 64°42'59"W, lower intertidal area, sand and algae growing on mangrove roots, 18 October 2011.

##### Type specimen.

Holotype: male, preserved in pure ethanol, deposition: Naturhistorisches Museum Wien, collection nr. NHMW 21884. Paratypes: two males, deposition: Senckenberg Museum für Naturkunde Görlitz, collection Nr. 11/48677.

##### Diagnosis.

Red-brown sclerotized mites. Average length 228 µm, mean width 139 µm. Notogaster rounded in dorsal view, hunchbacked in lateral view. A large anteriorly arched depression on anterior part of notogaster. Lamellar ridges short. Interlamellar seta of normal length and spiniform. Fourteen pairs of spiniform notogastral setae. Uniform median epimeral cavity. Three pairs of genital, two pairs of adanal and two pairs of anal setae present. Legs monodactylous; claw of each leg large. Claw with one proximoventral and a proximodorsal tooth. No porose areas on femora discernable.

##### Description.

Adult: Females (N=14), length: 222–244 μm (mean 231 μm), width: 136–152 µm (mean 142 µm); males (N=16), length: 212–238 μm (mean 226 μm), width: 131–147 µm (mean 137 µm)

Integument. Colour red brown. Cerotegument appears basically slightly granular. Cerotegument of prodorsum nearly smooth between bothridia, strongly granular anterior and lateral to lamellar ridges. Cerotegument of notogaster and venter slightly granular. Cerotegument of lateral body parts generally finely granular, larger granules in areas surrounding acetabula. Cerotegument of legs slightly granular.

Prodorsum. Rostrum rounded in dorsal view, but slightly projecting anteroventrally in lateral view. Rostral setae (*ro*) short and smooth. Lamellar setae (*le*) and interlamellar setae (*in*) simple, short and smooth. Exobothridial setae (*ex*) minute. Lamellar ridge conspicuous, but short, not reaching insertions of lamellar setae. Bothridium large cup exhibiting a strongly projecting posterior ridge with three lobe-like protrusions overhanging anterior border of gastronotic region. Sensillus long (ca. 50µm) and flagelliform. Tutorium developed as slightly dorsally curved ridge.

**Figure 1.**
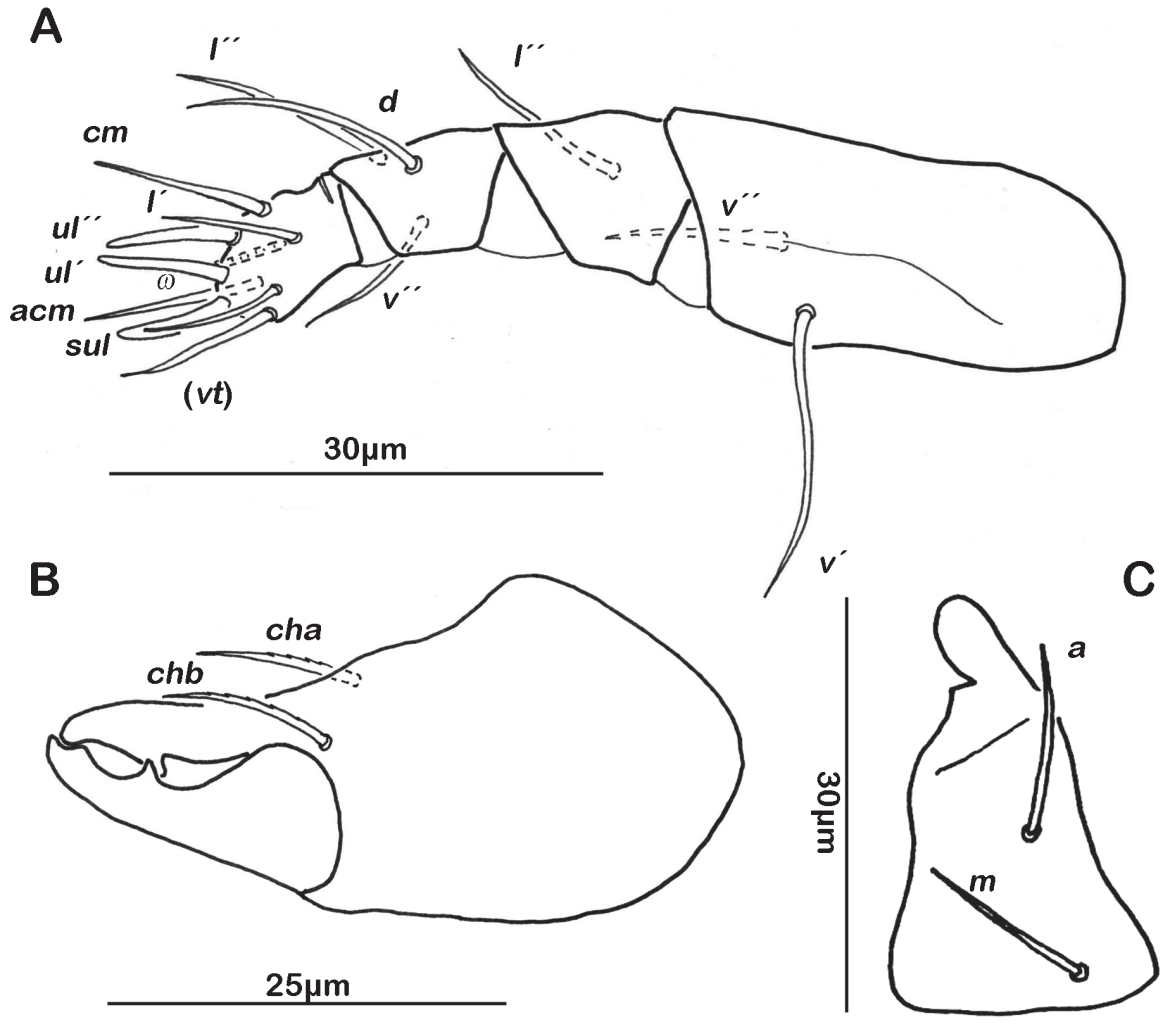
*Selenoribates quasimodo* sp. n. mouthparts. **A** left pedipalp antiaxial view **B** left chelicera antiaxial view **C** right rutellum ventral view.

Gnathosoma. Pedipalp pentamerous 0-2-1-3-9 (including solenidion) (Fig. 1A). Solenidion erect, not fused with eupathidium *acm*. Chelicera chelate, in lateral view forceps-like and each digit with two teeth, whereas from frontal view most distal teeth split into two symmetrical teeth (Fig. 1B). No porose area on proximal part of fixed digit discernable. Seta *cha* and *chb* dorsally slightly pectinate, both same length. Distal part of rutellum developed as thin triangular slightly curved inward membrane (Fig. 1C). Setae *a* and *m* long and smooth. Mentum regular, setae *h* simple, thin and long.

**Figure 2.**
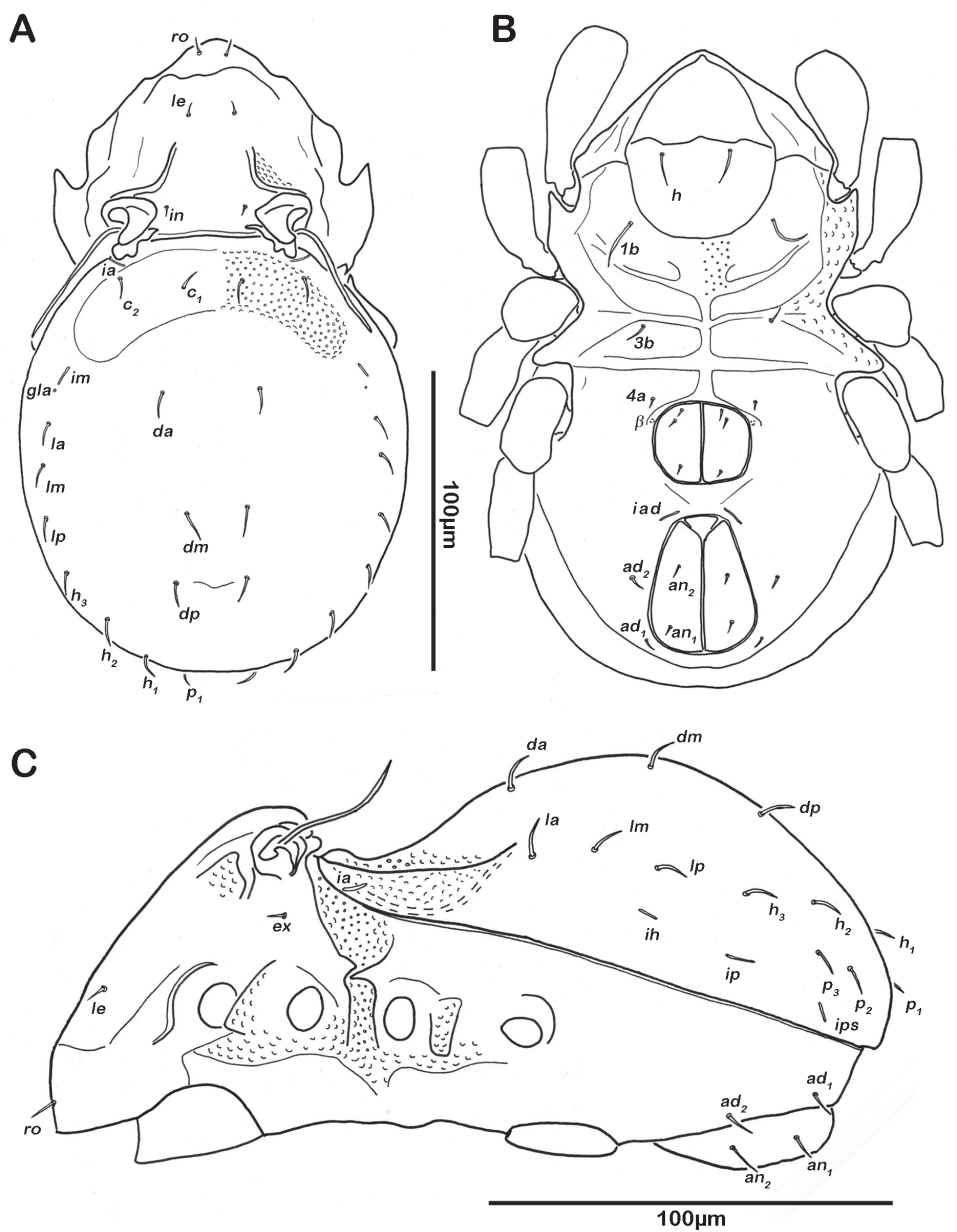
*Selenoribates quasimodo* sp. n. adult. **A** dorsal view **B** ventral view **C** lateral view.

**Figure 3.**
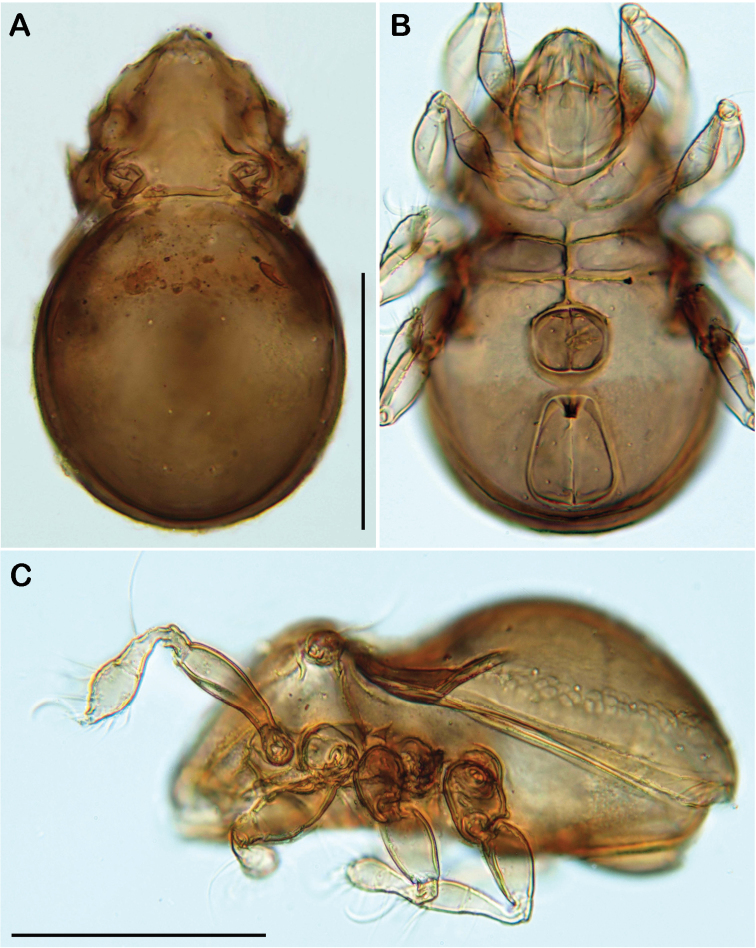
*Selenoribates quasimodo* sp. n. adult micrographs layered from 5–10 sequentially focused images. **A** dorsal view **B** ventral view **C** lateral view. Scale bars = 100 µm.

Notogaster (Figs 2A, 3A). Rounded in dorsal view, hunchbacked in lateral view. Anterior margin of notogaster distinct. A large, arched depression on anterior part of notogaster showing obvious granulation. Fourteen pairs of simple notogastral setae (approximate length 5–7 µm), *c_1-2_*, *da*, *dm*, *dp*, *la*, *lm*, *lp*, *h_1-3_*, *p_1-3_*; *c_3_* absent. Notogastral setae sometimes completely covered by a layer of cerotegument. Porose areas or distinct pores absent. Five pairs of notogastral lyrifissures present; *ia* next to setae *c_2_* close and rectangular to anterior notogastral border; *im* slightly anterior and laterad of setae *la*; lyrifissures *ih, ip* and *ips* laterally close to lateroventral borders of notogastral plate. Opisthonotal gland openings (*gla*) located posteriorly to lyrifissures *im*.

Lateral aspect (Figs 2C, 3C). Pedotectum I small but thick, pedotectum II absent. Lateral parts of anterior margin of notogaster broad and deep, showing conspicuous granules. Enantiophyse consisting of two strong triangular teeth orientated against each other. Discidium developed as strong rectangular bulge between acetabulum III and IV.

Ventral region of idiosoma (Figs 2B, 3B). Epimeral setation 1-0-1-1, seta *1b* long reaching trochanter II, setae *3b* normal length and *4a* short. Internal borders of all epimera well visible, sternal apodemes II, sejugal and III well developed. A densely granulated median sternal cavity on epimeron I. Three pairs of short and fine genital setae, arranged in longitudinal rows, anterior two pairs close to each other. Insertion of tendon *β* next to anterior corners of genital orifice. Aggenital setae absent. Anal plates triangular. Preanal organ triangular in ventral view. Two pairs of short anal setae, *an_1-2_*_,_ present. Two pairs of short and simple adanal setae *ad_1-2_* present, *ad_3_* absent. Lyrifissure *iad* obliquely, adjacent to anterior corners of anal orifice.

Legs. Monodactylous. Long hook-like claws with one conspicuous proximoventral and one minuscule proximodorsal tooth. Trochanters III and IV with an obvious triangular dorsodistal projection. Femora with slightly projecting ventral carinae. All tarsi with one proximal lyrifissure. No porose areas on femora discernable. Solenidia *φ_1_* on tibia I long and orientated backwards. Chaetome and Solenidia see Table 1.

##### Etymology.

The specific name refers to Quasimodo, the famous bell-ringer of Victor Hugo’s historical novel “Notre-Dame de Paris” (1831). This appellation is due to the hunchback of this species shown in lateral view (that does not necessarily mean the species is as ugly as the bell-ringer was supposed to be). The name is given as noun in apposition.

##### Juvenile instars - common features.

Apheredermous. Colour light brown. Integument strongly plicate, except for centrodorsal plate. Thick layer of cerotegument covering whole body. Prodorsum triangular, rostrum rounded. Rostral and lamellar setae short and smooth. Exobothridial setae reduced to a circular vestigial structure. Interlamellar setae very short. Sensillus long and flagelliform. Bothridium large cup, laterally opened. Gnathosoma no obvious differences to adult instar. Hysterosoma slightly concave, plateau-like. Slightly plicate centrodorsal plate occupying two thirds of dorsal hysterosoma, bearing centrodorsal setae. Hysterosomal cupules not discernable in any instar. Large folds framing centrodorsal plate completely, showing fine granular surface. Orifice of opisthonotal gland laterad of seta *ad_2_*. Integument surrounding anogenital area folded. Dorsal setae of tibiae and genua absent. No porose areas detectable in any stage.

##### Protonymph.

Length (N=3): 172–209 μm (mean 191 μm)

**Figure 4.**
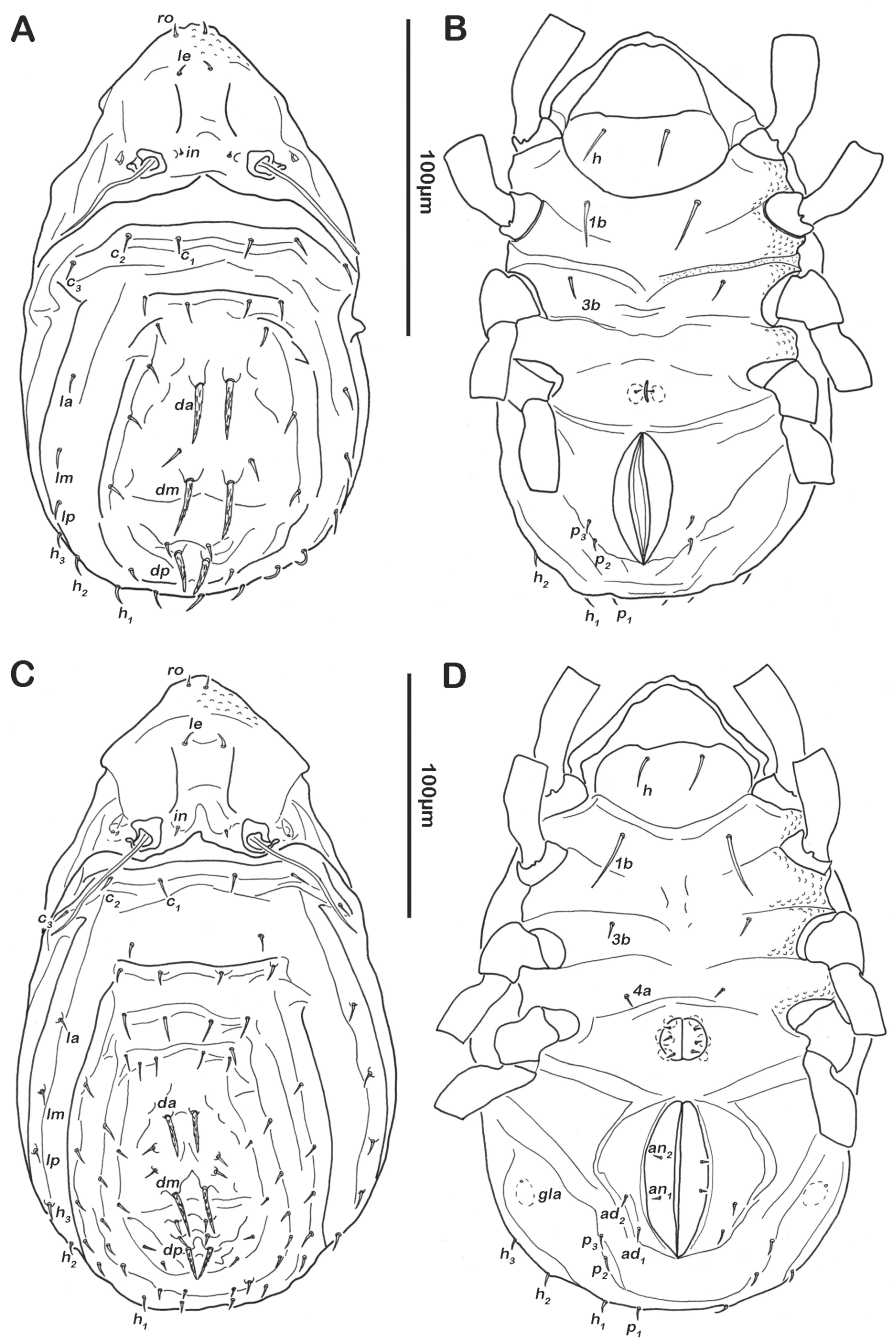
*Selenoribates quasimodo* sp. n. nymphs. **A** protonymph dorsal view **B** protonymph ventral view **C** tritonymph dorsal view **D** tritonymph ventral view.

Gastronotic region (Fig. 4A) with 24 pairs of notogastral setae; setae *c_1-3_*, *da*, *dm*, *dp*, *la*, *lm* and *lp* duplicated, *h_1-3_* and *p_1-3_* normal. Centrodorsal setae *da*, *dm* and *dp* robust and dorsally serrate, all other setae simple and small.

Ventral region of idiosoma (Fig. 4B). Epimeral setation 1-0-1-0. One pair of short genital setae.

Legs. Chaetome and Solenidia see Table 1.

**Table 1.** Chaetome and solenidia of the new Selenoribates species. Presence of setae characterized by letters. () = pairs of setae, – = no change with regard to preceding stage.<br/>

*Selenoribates quasimodo* sp. n.	instars	trochanter	femur	genu	tibia	tarsus	chaetome	solenidia
Leg I	protonymph	-	*d*, *d*, *l´*	(*l*), σ	(*l*), *ν´*, *φ_1_*	(*pv*), (*pl*), *s*, (*a*), (*u*), (*p*), (*tc*), (*ft*), ω *_1_*, ω *_2_*, ε	0-3-2-3-16	1-1-2
	tritonymph	-	-	-	*φ_2_*	(*it*)	0-3-2-3-18	1-2-2
	adult	-	-	-	-	-	0-3-2-3-18	1-2-2
Leg II	protonymph	-	*d*, *l"*	(*l*), σ	*l´*, *ν´*, *φ*	(*pv*), *s*, (*a*), (*u*), (*p*), (*tc*), (*ft*), ω	0-2-2-2-13	1-1-1
	tritonymph	-	*d*, *l´*	-	-	(*it*)	0-4-2-2-15	1-1-1
	adult	-	-	-	*l"*	-	0-4-2-3-15	1-1-1
Leg III	protonymph	-	*d*, *l´*	*l´*, σ	*l´*, *φ*	(*pv*), *s*, (*a*), (*u*), (*p*), (*tc*), (*ft*)	0-2-1-1-13	1-1-0
	tritonymph	-	-	-	-	-	0-2-1-1-13	1-1-0
	adult	*ν´*	-	-	*v"*	-	1-2-1-2-13	1-1-0
Leg IV	protonymph	-	-	-	-	(*pv*), (*u*), (*p*), *ft*´	0-0-0-0-7	0-0-0
	tritonymph	-	*d*, *l´*	*l´*	*l´*, *ν", φ*	*s*, (*a*), (*tc*)	0-2-1-2-12	0-1-0
	adult	*ν´*	-	-	-	-	1-2-1-2-12	0-1-0
*Selenoribates satanicus* sp. n.	instars	trochanter	femur	genu	tibia	tarsus	chaetome	solenidia
Leg I	adult	-	(*l*), *d*	(*l*), σ	(*l*), *ν´*, *φ_1,_ φ_2_*	(*pv*), (*pl*), *s*, (*a*), (*u*), (*p*), (*it*), (*tc*), (*ft*), ω *_1_*, ω *_2_*, ε	0-3-2-3-18	1-2-2
Leg II	adult	-	*d*, *d*, *l"*	(*l*), σ	(*l*), *ν´*, *φ*	(*pv*), *s*, (*a*), (*u*), (*p*), (*it*), (*tc*), (*ft*), ω	0-3-2-3-15	1-1-1
Leg III	adult	*ν´*	*d*, *l´*	*l´*, σ	*l*´, *ν´*, *φ*	(*pv*), *s*, (*a*), (*u*), (*p*), (*tc*), (*ft*)	1-2-1-2-13	1-1-0
Leg IV	adult	*ν´*	*d*, *l´*	*l´*	*l´*, (*ν*), *φ*	(*pv*), *s*, (*a*), (*u*), (*p*), (*tc*), *ft´*	1-2-1-3-12	0-1-0
*Selenoribates elegans* sp. n.	Instars	trochanter	femur	genu	tibia	tarsus	chaetome	solenidia
Leg I	adult	-	(*l*), *d*	(*l*), σ	(*l*), *ν´*, *φ_1,_ φ_2_*	(*pv*), (*pl*), *s*, (*a*), (*u*), (*p*), (*it*), (*tc*), (*ft*), ω *_1_*, ω *_2_*, ε	0-3-2-3-18	1-2-2
Leg II	adult	-	*d*, *d*, *l"*	(*l*), σ	(*l*), *ν´*, *φ*	(*pv*), *s*, (*a*), (*u*), (*p*), (*it*), (*tc*), (*ft*), ω	0-3-2-3-15	1-1-1
Leg III	adult	*ν´*	*d*, *l´*	*l´*, σ	*l*´, *ν´*, *φ*	(*pv*), *s*, (*a*), (*u*), (*p*), (*tc*), (*ft*)	1-2-1-2-13	1-1-0
Leg IV	adult	*ν´*	*d*, *l´*	*l´*	*l´*, (*ν*), *φ*	(*pv*), *s*, (*a*), (*u*), (*p*), (*tc*), *ft´*	1-2-1-3-12	0-1-0

##### Tritonymph.

Length (N=9): 228–266 μm (mean 243 μm)

Gastronotic region (Fig. 4C, 5A). 44 pairs of notogastral setae, setae of series *c*, *d*, *l* and *h*-series further multiplied.

Ventral region of idiosoma (Fig. 4D, 5B). Epimeral setation 1-0-1-1. Three pairs of short genital setae in a longitudinal row. Two pairs of adanal setae *ad_1-2_*. Two pairs of anal setae *an_1-2_*.

Legs (Fig. 6). Chaetome and Solenidia see Table 1.

**Figure 5.**
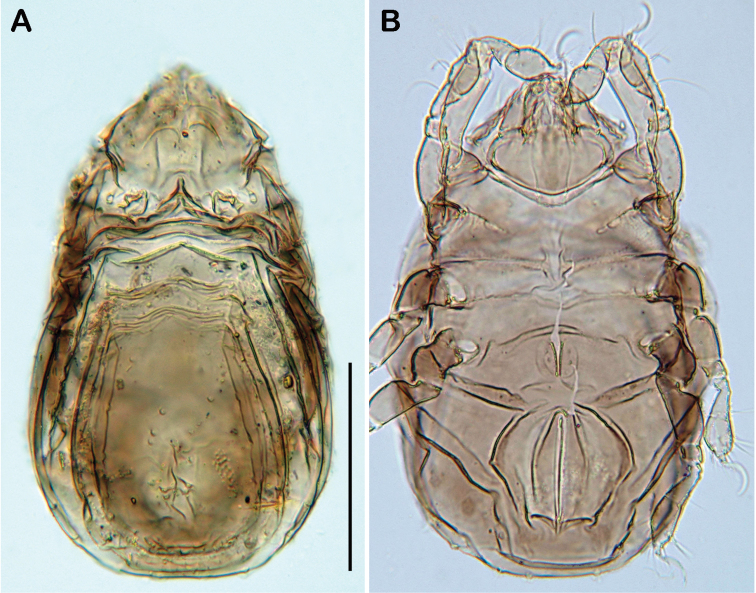
*Selenoribates quasimodo* sp. n. tritonymph, micrographs layered from 5–10 sequentially focused images. **A** dorsal view **B** ventral view. Scale bar = 100 µm.

**Figure 6.**
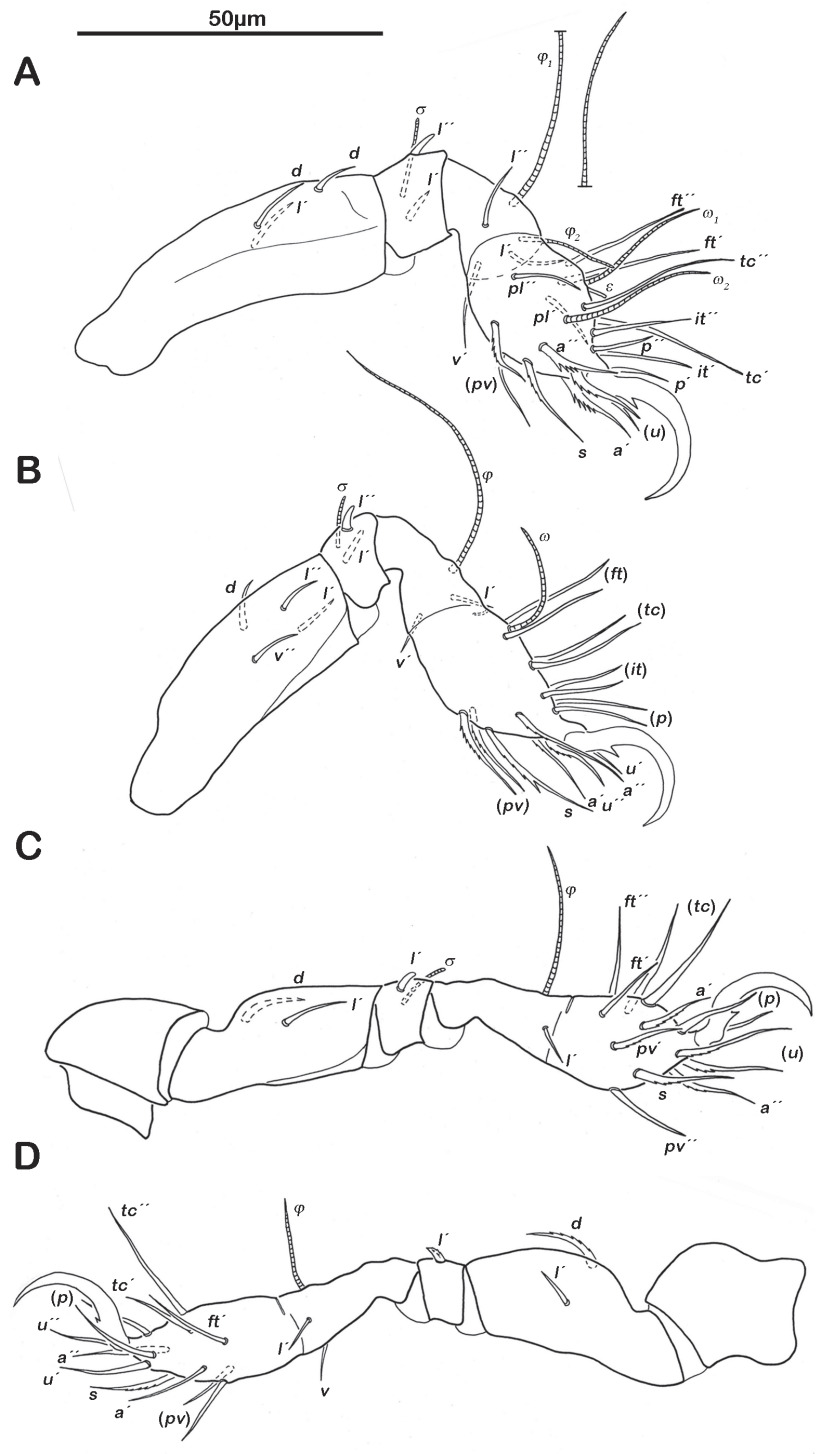
*Selenoribates quasimodo* sp. n. tritonymph legs. **A** right leg I antiaxial view **B** right leg II antiaxial view **C** left leg III antiaxial view **D** right leg IV antiaxial view.

#### Selenoribates satanicus sp. n.

urn:lsid:zoobank.org:act:30EC290D-FD6A-4E7B-B581-30F627ADC954

http://species-id.net/wiki/Selenoribates_satanicus

##### Type locality.

Bermuda: Lover’s Lake (marine pond), 32°22'03"N, 64°42'37"W, median eulittoral zone, green algae growing on sandy substrate among mangrove roots, 26 January 2012.

##### Type specimen.

Holotype: female, preserved in pure ethanol, deposition: Naturhistorisches Museum Wien, collection nr. NHMW 21885. Paratypes: 1 female and 1 male, same locality as holotype; deposition: Senckenberg Museum für Naturkunde Görlitz, collection Nr. 12/48678.

##### Diagnosis.

Light brown sclerotized mites. Average length 283 µm, mean width 174 µm. Notogaster rounded in dorsal view, concave in lateral view. Lamellar ridges long reaching lamellar setae. On anterior border of notogaster a pair of horn-like structures. Three notogastral depressions, framed by longitudinal ridges. Fourteen pairs of spiniform notogastral setae. Two median epimeral cavities. Three pairs of genital setae. Three pairs of adanal and one pair of anal setae. Legs monodactylous with large claw. Claw with one proximoventral and one proximodorsal tooth.

##### Description.

Adult: Females (N=6), length: 278–308 μm (mean 290 μm), width: 175–188 µm (mean 181 µm); males (N=5), length: 265–291 μm (mean 274 μm), width: 163–172 µm (mean 168 µm).

Integument. Colour light brown. Cuticle appears granular under dissecting microscope. Cerotegument of prodorsum and notogaster granular. Cerotegument of lateral parts generally finely granular, larger granules in areas surrounding acetabula. Ventral cerotegument generally finely granular, areas laterad of anal opening showing stronger granulation.

Prodorsum. Rostrum rounded in dorsal view, but slightly projecting anteroventrally in lateral view. Rostral setae (*ro*), lamellar setae (*le*) and interlamellar setae (*in*) short and simple. One pair of minute exobothridial setae (*ex*). Lamellar ridges conspicuous, reaching insertions of lamellar setae. Bothridium large cup with a projecting posterior ridge. Sensillus long (ca. 53 µm) and flagelliform. Tutorium weakly developed and small.

Gnathosoma. Pedipalp pentamerous 0-2-1-3-9 (including solenidion). Solenidion erect, not fused with eupathidium *acm*. Chelicera chelate, in lateral view forceps-like and each digit with two teeth, whereas from frontal view most distal teeth split into two symmetrical teeth. Distal part of rutellum developed as thin triangular slightly curved inward membrane. Setae *a* and *m* long and smooth. Mentum regular, setae *h* simple, thin and long.

**Figure 7.**
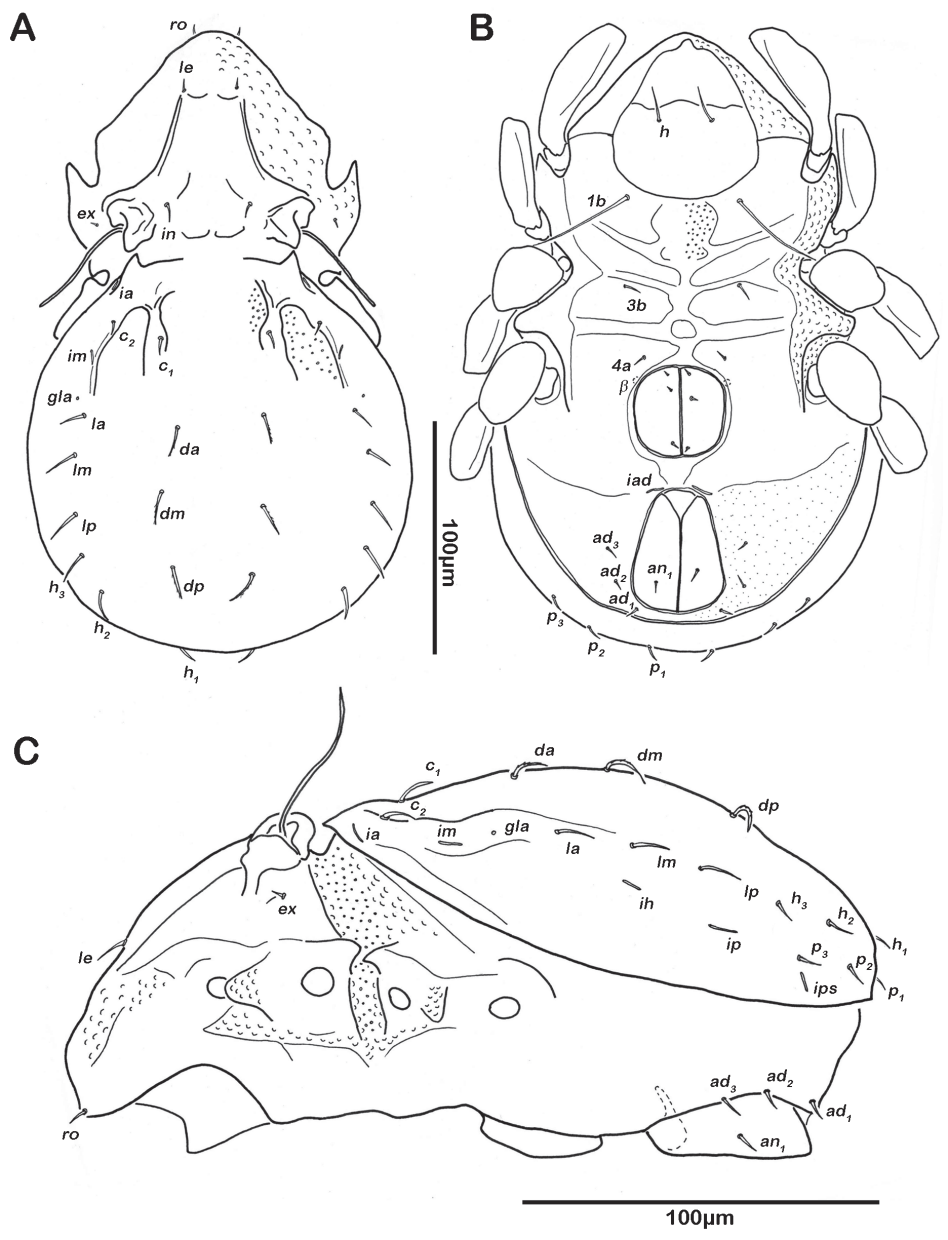
*Selenoribates satanicus* sp. n. adult. **A** dorsal view **B** ventral view **C** lateral view.

**Figure 8.**
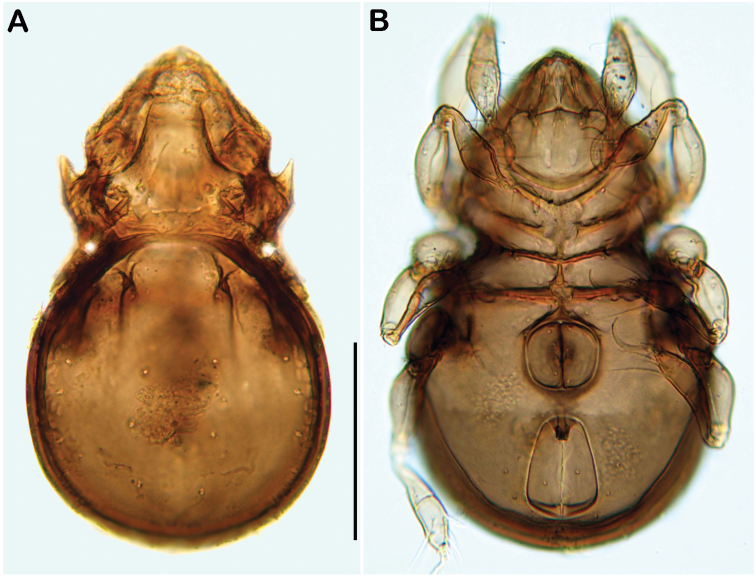
*Selenoribates satanicus* sp. n. adult micrographs layered from 5–10 sequentially focused images. **A** dorsal view **B** ventral view. Scale bar = 100 µm.

Notogaster (Figs 7A, 8A). Rounded in dorsal view, concave in lateral view. Anterior margin of notogaster incomplete, medially interrupted. On anterior border of notogaster a pair of strongly anteriorly projecting horn-like structures, situated directly posterior of bothridia. Three notogastral depressions on anterior third of notogaster, framed by longitudinal ridges reaching transversal line of setae *la* and *da*. Cerotegument of depressions strongly granular. Fourteen pairs of notogastral setae, *c_1-2_*, *da*, *dm*, *dp*, *la*, *lm*, *lp*, *h_1-3_*, *p_1-3_* (approximate length 10-13 µm); *c_3_* absent. Setae *da*-*dp*, slightly serrate, all other setae smooth. Porose areas or distinct pores absent. Five pairs of notogastral lyrifissures present; *ia* anterior to seta *c_2_*, in dorsal view hidden under anterior notogastral horn-like projection; *im* posterior of seta *c_2_*; lyrifissures *ih, ip* and *ips* laterally close to lateroventral border of notogastral plate. Orifice of opisthonotal gland (*gla*) next to setae *la*.

Lateral aspect (Figs 7C, 8C). Pedotectum I small but thick, pedotectum II absent. Lateral sejugal furrow broad and deep, showing conspicuous granulation. Enantiophyse consisting of two strong triangular teeth orientated against each other. Anterior tooth slightly rounded. Discidium developed as strong triangular bulge between acetabulum III and IV.

Ventral region of idiosoma (Figs 7B, 8B). Epimeral setation 1-0-1-1, seta *1b* long reaching trochanter III, setae *3b* and *4a* short. Internal borders of all epimera well visible, sternal apodemes II, sejugal and III well developed. A densely granulated median sternal cavity on epimeron I and a second circular median cavity on a level with apodemes III. Three pairs of short and fine genital setae, arranged in longitudinal rows, anterior two pairs of setae close to each other. Insertion of tendon *β* next to anterior corners of genital orifice. Aggenital setae absent. Anal valves triangular. Preanal organ shaped triangular in ventral view. One pair of short anal setae, *an_1_*, located on posterior half of anal valves. Three pairs of short and simple adanal setae *ad_1-3_*. Lyrifissure *iad* obliquely, adjacent to anterior corners of anal orifice.

**Figure 9.**
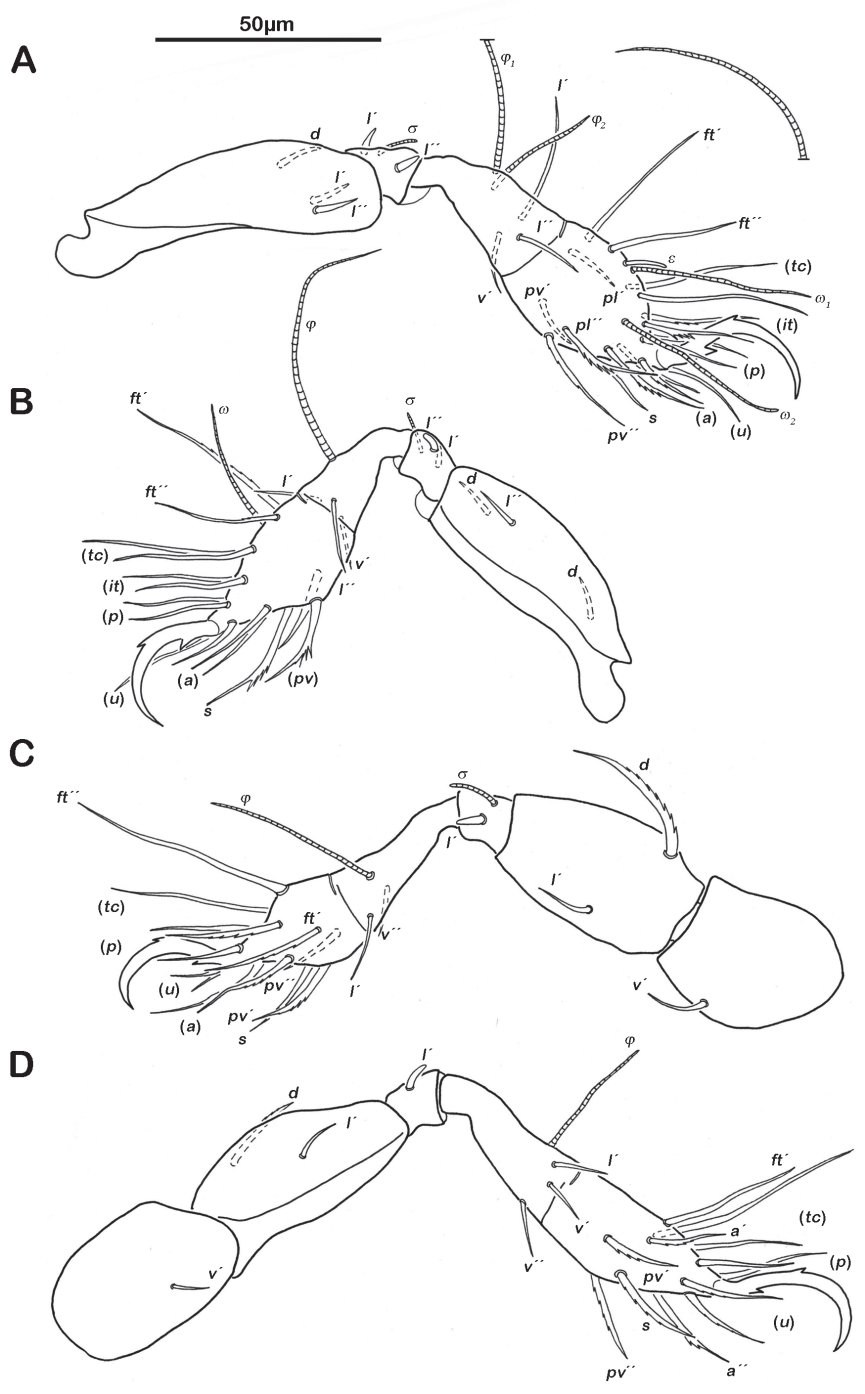
*Selenoribates satanicus* sp. n. legs. **A** right leg I antiaxial view **B** right leg II axial view **C** right leg III antiaxial view **D** left leg IV antiaxial view.

Legs (Fig. 9). Monodactylous. Long pointed hook-like claw with one conspicuous proximoventral and a minute proximodorsal tooth. Trochanters III and IV with a triangular dorsodistal projection. Femora exhibiting slightly projecting ventral carinae. All tarsi with one proximal lyrifissure. No porose areas on femora discernable. Solenidion *φ_1_* on tibia I long, orientated backwards. Chaetome and Solenidia see Table 1.

Larva. Length (N=1): 137 μm

**Figure 10.**
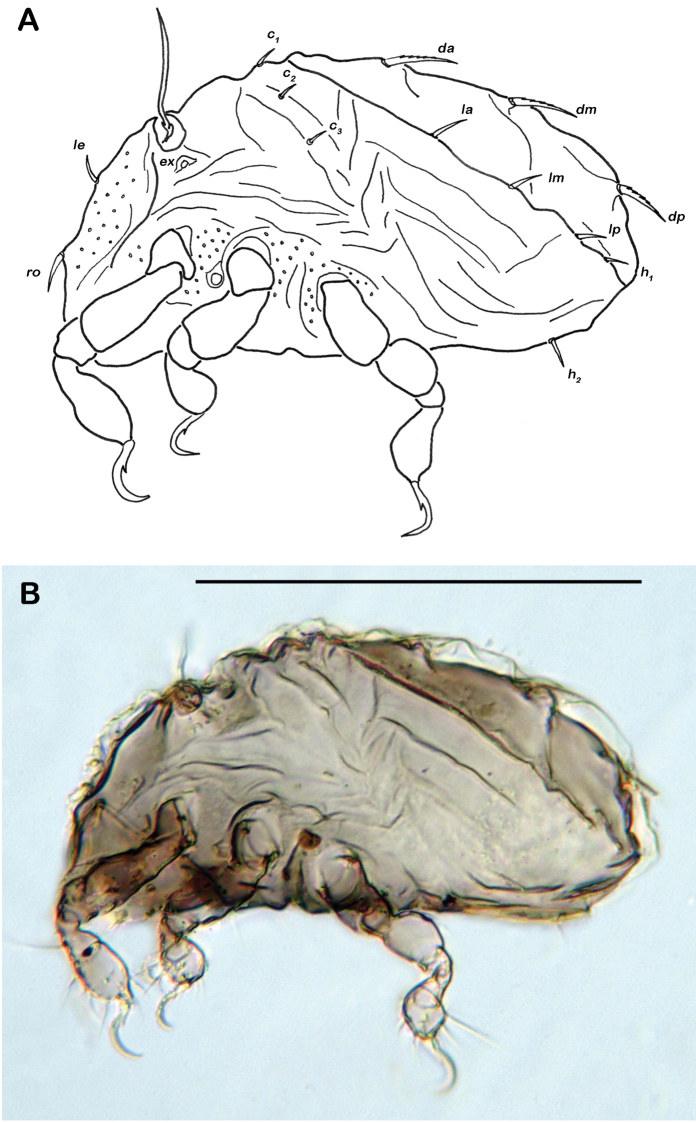
*Selenoribates satanicus* sp. n. larva. **A** lateral view **B** lateral view, micrograph layered from 5–10 sequentially focused images. Scale bar = 100 µm.

Gastronotic region (Fig. 10). 11 pairs of notogastral setae; setae *c_1-3_*, *da*, *dm*, *dp*, *la*, *lm*, *lp* and *h_1-2_*, *h_3_* absent. Centrodorsal setae *da*, *dm* and *dp* robust and dorsally serrate, all other setae simple and small.

Ventral region of idiosoma. Epimeral setation 1-0-1. Claparède organ bladder-like. No protecting seta detectable.

##### Etymology.

When the author regarded *Selenoribates satanicus* in dorsal view for the first time, the oval contour of the notogaster with its two anterior horn-like projections reminded him of the silhouette of the devil’s face, therefore the specific name refers to the Hebraic name Satan and is given as adjective in the nominative singular.

#### Selenoribates elegans sp. n.

urn:lsid:zoobank.org:act:F9258443-DBA2-408F-890D-2DC72CA9938D

http://species-id.net/wiki/Selenoribates_elegans

##### Type locality.

Bermuda, Whalebone Bay, 32°21'55"N, 64°42'49"W, lower intertidal area, red algae on rocks, 22 November 2011.

##### Type specimen.

Holotype: female, preserved in pure ethanol, deposition: Naturhistorisches Museum Wien, collection nr. NHMW 21886.

##### Diagnosis.

Red-brown sclerotized mites. Average length 202 µm, mean width 115 µm. Notogaster oval in dorsal view, slightly concave in lateral view. Lamellar ridges absent. Interlamellar setae normal and minute. Two X-shaped ridges on anterior part of notogaster. Fourteen pairs of simple long notogastral setae. Two median epimeral cavities. Claw with two proximoventral and one proximodorsal tooth.

##### Description.

Adult: Males (N=2), length: 200–203 μm (mean 201.5 μm), width: 108–122 µm (mean 115 µm).

Integument. Colour light brown. Cuticle showing dotted pattern. Cerotegument of notogaster granular, larger granules in centre of gastronotic plate. Cerotegument of lateral parts granular, with larger granulation in areas surrounding acetabula. Ventral region finely granular, denser granulation laterad of anal orifice.

Prodorsum. Cerotegument strongly granular. Rostrum rounded in dorsal view, but slightly projecting anteroventrally in lateral view. Rostral (*ro*) and lamellar setae (*le*) simple and short, interlamellar setae (*in*) very short. One pair of minute exobothridial setae (*ex*). Lamellar ridges absent. Bothridium large cup without posterior ridge. Sensillus long (ca. 48 µm), flagelliform.

Gnathosoma. Pedipalp pentamerous 0-2-1-3-9 (solenidion included). Solenidion erect, not fused with eupathidium *acm*. Chelicera chelate, forceps-like in lateral view, each digit with two teeth, whereas from frontal view most distal teeth split into two symmetrical teeth. Distal part of rutellum a thin triangular slightly inward curved membrane. Setae *a* and *m* long and smooth. Mentum regular, setae *h* simple, thin and long.

**Figure 11.**
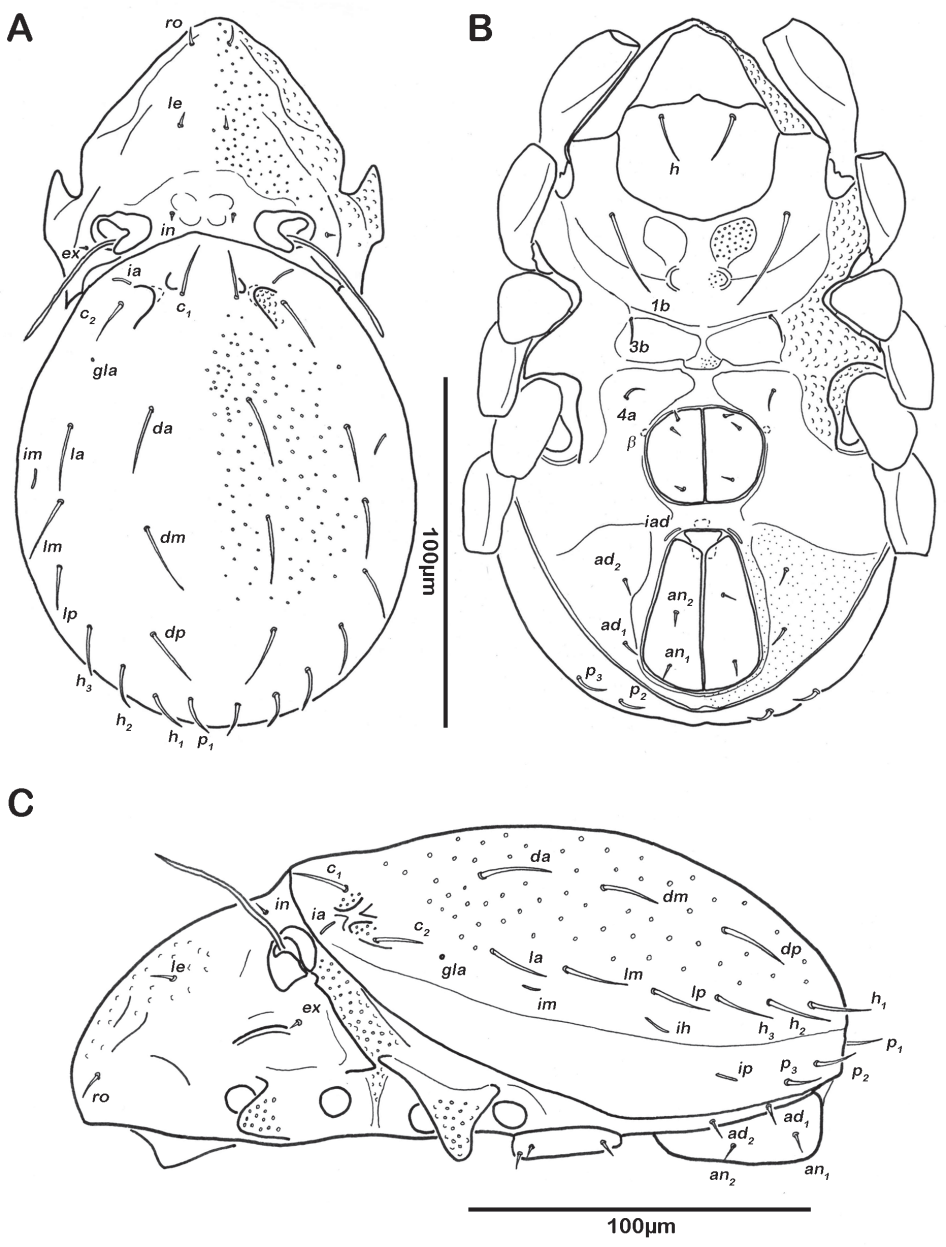
*Selenoribates elegans* sp. n. adult. **A** dorsal view **B** ventral view **C** lateral view.

**Figure 12.**
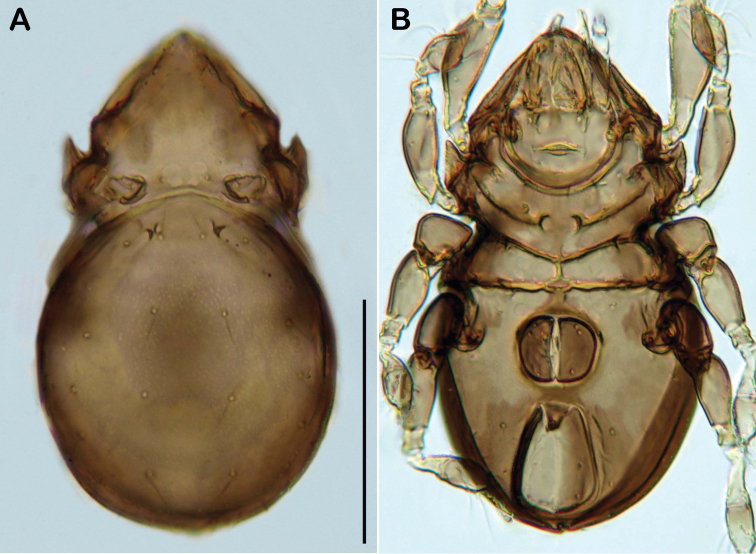
*Selenoribates elegans* sp. n. adult micrographs layered from 5–10 sequentially focused images. **A** dorsal view **B** ventral view. Scale bar = 100 µm.

Notogaster (Figs 11A, 12A). Rounded in dorsal view, slightly concave in lateral view. Anterior margin of notogaster complete. On anterior part of notogaster a pair of small X-shaped ridges, close to seta *c_1_*. Fourteen pairs of simple notogastral setae, *c_1-2_*, *da*, *dm*, *dp*, *la*, *lm*, *lp*, *h_1-3_*, *p_1-3_* (approximate length 17-20 µm), setae *c_3_* absent. Porose areas or distinct pores absent. Five pairs of notogastral lyrifissures present; *ia* anterior to seta *c_2_*; *im* posterior and laterad of seta *la*; lyrifissures *ih, ip* and *ips* laterally close to lateroventral border of notogastral plate. Orifice of opisthonotal gland (*gla*) posterior to seta *c_2_*.

Lateral aspect. Pedotectum I small but robust, pedotectum II absent. Enantiophyse consisting of two strong triangular and pointed teeth orientated against each other. Discidium developed as strong triangular projection between acetabulum III and IV.

Ventral region of idiosoma (Figs 11B, 12B). Epimeral setation 1-0-1-1, seta *1b* long reaching trochanter III, setae *3b* and *4a* of normal length and simple. Internal borders of all epimera well visible, sternal apodemes III and IV well developed. Median sternal cavity on epimeron I divided into two anterior symmetric parts and one unpaired posterior part, all parts strongly granulated. A second triangular median cavity on epimeron III on a level with apodeme 3. Three pairs of short and fine genital setae, arranged in longitudinal rows, anterior pairs close to each other. Insertion of tendon *β* adjacent to anterior corners of genital orifice. Aggenital setae absent. Anal plates triangular. Preanal organ triangular. Two pairs of short anal setae, *an_1-2_*. Two pairs of short and simple adanal setae *ad_1-2_*, *ad_3_* absent. Lyrifissure *iad* obliquely, next to anterior corners of anal valves.

Legs. Monodactylous. Long acute hook-like claw with two obvious proximoventral teeth, one close to the base of claw and one proximodorsal tooth. Cuticle finely granular. Femora with projecting ventral carinae. All tarsi with one proximal lyrifissure. Porose areas absent. Solenidia *φ_1_* on tibia I long, orientated backwards. Chaetome and solenidia see Table 1.

##### Etymology.

The specific name is derived from the Latin word *elegans* meaning elegant and refers to the slender and delicate shape of the whole body. The name is given as adjective in the nominative singular.

### Key to the *Selenoribates* species

**Table:** 

1	Three depressions on anterior part of notogaster separated by two X-shaped ridges	2
–	Depressions and ridges shaped different	5
2	Three pairs of anal, four pairs of genital setae	*Selenoribates ghardaqensis* Abd-El-Hamid, 1973
–	Two pairs of anal setae and three pairs of genital setae	3
3	Sensillus spatuliform	*Selenoribates mediterraneus* Grandjean, 1966
–	Sensillus flagelliform	4
4	Anterior median epimeral cavity simple and shaped circular; lamellar ridges short but conspicuous	*Selenoribates foveiventris* Strenzke, 1961
–	Anterior median epimeral cavity divided into two anterior symmetric parts and one unpaired posterior part; lamellar ridges absent	*Selenoribates elegans* sp. n.
5	Three depressions on anterior part of notogaster separated by longitudinal ridges reaching transversal line of setae *la* and *da*; on anterior border of notogaster a pair of horn-like projections	*Selenoribates satanicus* sp. n.
–	A single large depression on anterior part of notogaster, causing a hunchbacked appearance in lateral view	*Selenoribates quasimodo* sp. n.

## Discussion

**Figure 13.**
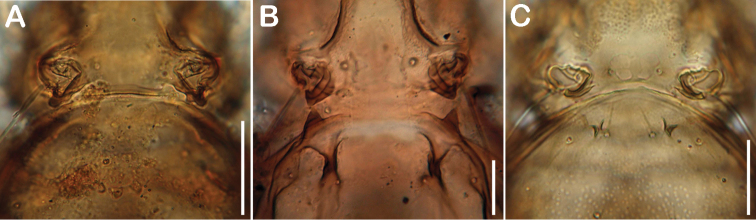
Comparison of cuticular structures on anterior part of gastronotic region. **A** *Selenoribates quasimodo* sp. n. **B** *Selenoribates satanicus* sp. n. **C** *Selenoribates elegans* sp. n. Scale bars = 30 µm.

**Figure 14.**
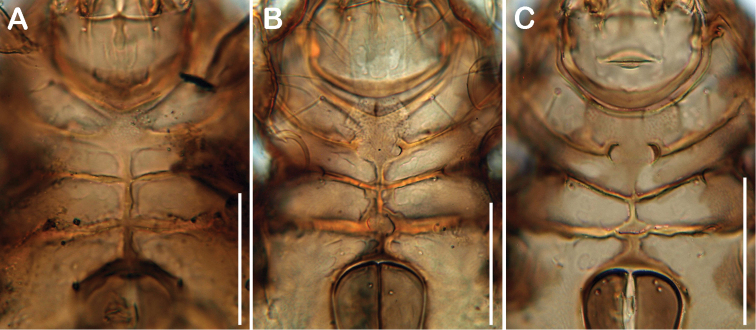
Comparison of ventral cavities. **A** *Selenoribates quasimodo* sp. n. **B** *Selenoribates satanicus* sp. n. **C** *Selenoribates elegans* sp. n. Scale bars 50 µm.

Grandjean (1966) mentioned three depressions separated by two X-shaped ridges on the anterior part of the gastronotic region as diagnostic character of the genus *Selenoribates*. The new species possess anterior notogastral depressions and sometimes ridges, but they differ in shape and size (Fig. 13). Especially *Selenoribates quasimodo* sp. n. diverges in this respect, showing a single large deepening without any ridges. Notogastral depressions may represent a synapomorphic character of this genus, but the detailed configuration has evolved in different ways. Moreover, Pfingstl and Schuster (2012) described *Carinozetes trifoveatus*, another selenoribatid species, also exhibiting three anterior gastronotic depressions and two X-shaped ridges, similar to that shown in most of the *Selenoribates* species. As *Carinozetes trifoveatus* is subject to the same selective constraints of the littoral environment, this character may have evolved convergently. However, median epimeral cavities are also present in *Carinozetes* Pfingstl & Schuster, 2012 and in *Thalassozetes riparius* Schuster, 1963, but the possession of two cavities is unique to the genus *Selenoribates*. Accordingly, this character state represents another synapomorphy of this taxon, whereas the specific shape of the cavities varies among species (Fig. 14), and hence represents a valuable trait for species discrimination. Comparing the new species with the already known members of *Selenoribates*, one interesting fact becomes obvious (Table 2). *Selenoribates elegans* sp. n. shows conformity in most of its morphological features with *Selenoribates foveiventris*, *Selenoribates mediterraneus* and *Selenoribates ghardaqensis*, whereas *Selenoribates quasimodo* sp. n. and *Selenoribates satanicus* sp. n. deviate conspicuously from the others as well as from each other. Although *Selenoribates elegans* sp. n. was found on Bermuda, together with the latter species, its morphology suggests that it is closer related to the species from the Mediterranean and the Red Sea. *Selenoribates quasimodo* sp. n. and *Selenoribates satanicus* sp. n. with their more complex morphological features, on the other hand, may represent members of another lineage within the genus *Selenoribates*. However, the new species are unambiguous members of *Selenoribates* and based on their morphology, the genus diagnosis provided by Grandjean (1966) should be slightly adjusted as it was done here in the results part.

**Table 2.** Comparison of diagnostic morphological features of all Selenoribates species. ? = no information available; depr. = depression; X = X-shaped ridge.<br/>

	***Selenoribates foveiventris***	***Selenoribates mediterraneus***	***Selenoribates ghardaqensis***	***Selenoribates quasimodo***	***Selenoribates satanicus***	***Selenoribates elegans***
size (µm)	240-250	242-251	198-218	212-244	265-308	200-203
exobothridial seta	vestigial	minute	?	minute	minute	minute
lamellar ridges	short	short	short	short	long	absent
gastronotic structures	3 depr. 2X	3 depr. 2X	3 depr. 2X	1 depr.	3 depr.	3 depr. 2X
notogastral setae	14	14	14	14	14	14
epimeral setae	1-0-1-1	1-0-1-1	1-0-1-1	1-0-1-1	1-0-1-1	1-0-1-1
genital setae	3	3	4	3	3	3
anal setae	2	2	3	2	1	2
adanal setae	2	2	2	2	3	2
claw / ventral teeth	2	2	2	1	1	2

Knowledge about juvenile morphology of this taxon is largely incomplete and only Grandjean (1966) gave a detailed description of the deuto- and tritonymph of *Selenoribates mediterraneus*. Unfortunately, only the proto- and tritonymph of *Selenoribates quasimodo* sp. n. and the larva of *Selenoribates satanicus* sp. n. were available for the present study, so a comprehensive comparison and analysis of the ontogeny is not feasible. Nevertheless, the instars of *Selenoribates quasimodo* sp. n. and *Selenoribates satanicus* sp. n. posses a centrodorsal plate framed by lateral and ventral folds, the typical habitus of selenoribatid juveniles, and further exhibit conformity in most aspects with the nymphs of *Selenoribates mediterraneus*, confirming the familial and generic relationship of the species. Besides that, the nymphs of *Selenoribates quasimodo* sp. n. show an interesting case of ontogenetic neotrichy with most of the gastronotic setae being duplicated with each moult so that their number increases from stage to stage. This type of neotrichy should be classified as a cosmiotrichy, as the placement of secondary setae follows a distinct arrangement (van der Hammen 1981). Neotrichy shown in juvenile stages is a rare but not unknown phenomenon in oribatid mites. Nymphs of *Hydrozetes parisiensis* exhibit secondary notogastral setae (Grandjean 1948), the juveniles of *Tricheremaeus nemossensis* also show neotrichy (Grandjean 1963) and the number of secondary setae is increasing from stage to stage in the lohmanniid *Annectacarus mucronatus* (Grandjean 1950). But in all these examples the neotrichy persists throughout the adult stage, whereas in *Selenoribates quasimodo* sp. n. all the secondary setae get lost in the adults. However, to clarify the nature and the occurrence of this phenomenon in the genus *Selenoribates* it is necessary to investigate the complete development of all species.

**Figure 15.**
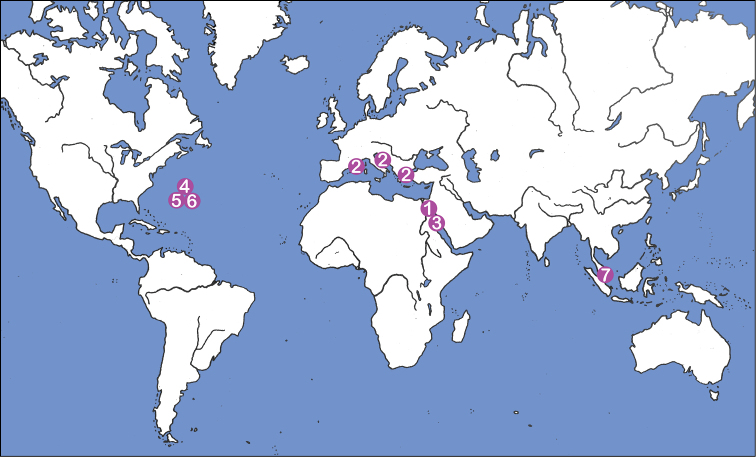
Map showing world distribution of the genus *Selenoribates*: **1** *Selenoribates foveiventris*, Egypt (Strenzke 1961) **2** *Selenoribates mediterraneus*, France, Croatia, Greece (Grandjean 1966) **3** *Selenoribates ghardaqensis*, Egypt (Abd-El-Hamid 1973) **4** *Selenoribates quasimodo* sp. n., Bermuda **5** *Selenoribates satanicus* sp. n., Bermuda **6** *Selenoribates elegans* sp. n., Bermuda **7** *Selenoribates* sp., Singapore (leg. Ilse Bartsch).

The biogeographic distribution of the genus *Selenoribates* was formerly limited to the Mediterranean and the Red Sea, but the records of new species from Bermuda clearly demonstrate that members of this genus also exist on coasts of the Western Atlantic. Moreover, littoral samples from Singapore, kindly provided by Ilse Bartsch, also contained specimens of a yet undescribed *Selenoribates* species. These new findings suggest that members of this genus show a much wider distribution than formerly supposed (Fig. 15) probably occurring on most coasts of tropic and subtropic regions. However, not only the biogeography, but also the diversity of *Selenoribates* must be reconsidered based on the present data. Bermuda is, with ca. 55 km^2^, one of the smallest countries of the world and harbours just as much *Selenoribates* species as the whole Mediterranean and the Red Sea together. Of course the Bermudian intertidal mite fauna may be derived from the Caribbean region and the species found on Bermuda may show a much wider distribution, but this clearly indicates that the real number of species may exceed the presently documented number by far. Pfingstl and Schuster (2012) already stated that within the Caribbean area, with its numerous islands, a relatively high diversity of selenoribatid species should be assumed and the same may apply to many other similar geographic regions, as for example the Indo-pacific area. However, if the genus *Selenoribates* is much more diverse than formerly known, the question arises why only a few species have been discovered yet and there are several possible answers to this question. First, *Selenoribates* specimens are relatively small for oribatid mites and may be easily overlooked, second, the littoral environment has been sampled only marginally in matters of oribatid mites and third, it is still unclear which microhabitat within the intertidal zone is usually occupied by *Selenoribates* species. Even in the present study specimens of this genus were found infrequently and in very small abundances. Accordingly it was not possible to assess if these mites are specifically associated with rocky or sandy substrate or with a specific alga etc. Nevertheless, further studies should answer the question of ecological needs and preferences and maybe then it will be easier to sample *Selenoribates* and reveal further species.

## Supplementary Material

XML Treatment for Selenoribates

XML Treatment for Selenoribates quasimodo

XML Treatment for Selenoribates satanicus

XML Treatment for Selenoribates elegans

## Appendix

Table of localities. (doi: 10.3897/zookeys.312.5478.app) File format: Microsoft Excel file (xls). **Explanation note:** Detailed information on sampling localities of the three new Selenoribates species.
